# Dipyridamole potentiated the trypanocidal effect of nifurtimox and improved the cardiac function in NMRI mice with acute chagasic myocarditis

**DOI:** 10.1590/0074-02760160499

**Published:** 2017-09

**Authors:** Sonia Santeliz, Peter Caicedo, Elidiosmar Giraldo, Carmen Alvarez, María-Daniela Yustiz, Claudina Rodríguez-Bonfante, Romina Bonfante-Rodríguez, Rafael Bonfante-Cabarcas

**Affiliations:** 1 Decanato de Ciencias Veterinarias, Unidad de Biomedicina, Departamento de Medicina y Cirugía, Barquisimeto, Estado Lara, Venezuela; 2 Decanato de Ciencias de la Salud, Unidad de Bioquímica, Barquisimeto, Estado Lara, Venezuela; 3 Universidad Centroccidental Lisandro Alvarado, Unidad de Parasitología Médica, Barquisimeto, Lara, Venezuela

**Keywords:** Chagas disease, nifurtimox, dipyridamole, Trypanosoma cruzi, treatment

## Abstract

**BACKGROUND:**

As chronic Chagas disease does not have a definitive treatment, the development of alternative therapeutic protocols is a priority. Dipyridamole (DPY) is an alternative to counteract the pathophysiological phenomena involved in Chagas cardiomyopathy.

**OBJECTIVE:**

To evaluate the therapeutic efficacy of DPY associated with nifurtimox (Nfx) in epimastigote axenic cultures and in mice with acute Chagas disease.

**METHODS:**

NMRI adult male mice were divided into nine groups: three healthy and six *Trypanosoma cruzi*-infected groups. Mice received vehicle, Nfx or DPY, alone or combined. The doses assayed were Nfx 10 and 40 mg/kg and DPY 30 mg/kg. The treatment efficacy was evaluated by clinical, electrocardiographic, parasitological, biochemical and histopathological methods.

**FINDINGS:**

*In vitro*, DPY and Nfx had a trypanocidal effect with IC_50_ values of 372 ± 52 and 21.53 ± 2.13 µM, respectively; DPY potentiated the Nfx effect. *In vivo*, Nfx (40 mg/kg) with or without DPY had a therapeutic effect, which was reflected in the 84-92% survival rate and elimination of parasitaemia and heart tissue amastigotes. Nfx (10 mg/kg) had a subtherapeutic effect with no survival and persistence of amastigotes, inflammation and fibrosis in heart tissue; adding DPY increased the survival rate to 85%, and all tested parameters were significantly improved.

**MAIN CONCLUSION:**

DPY has a trypanocidal effect *in vitro* and enhances the Nfx therapeutic effect in an *in vivo* murine model.

In the last two decades, sufficient data have been generated to suggest that Chagas disease is a global public health problem. Approximately 6 million to 7 million people worldwide, mostly in Latin America, are estimated to be infected with *Trypanosoma cruzi*, the parasite that causes Chagas disease, which is found mainly in endemic areas of 21 Latin American countries, where it is estimated that there is a population of 70,199,360 individuals at risk of acquiring the infection; 5,742,167 infected people; and 29,925 new cases per year with a mortality of 12,000 individuals per year (Dias et al. 2016, WHO 2017). Active vector transmission remains, which is reflected in the onset of acute Chagas disease cases in regions infested by vectors (Añez et al. 2004), and there is re-emergence of the disease manifested by the appearance of circumscribed outbreaks by oral transmission (Alarcón de Noya et al. 2015). The migration of infected people from underdeveloped South American countries to developed countries has enabled disease globalization, reporting a prevalence of 4.2% in these immigrant Latin-American populations in those countries (Requena-Méndez et al. 2015).

Although Chagas disease vector transmission has been controlled in most endemic areas of Latin America with a drastic decrease in new cases, Chagas disease still has a high residual prevalence in areas such as Bolivia (Medrano-Mercado et al. 2008), and a high incidence of acute cases is present in specific areas (Añez et al. 2004, Alarcón de Noya et al. 2015). As parasitic treatment in chronic cases remains controversial due to a lack of clinical efficacy of the current trypanocidal drugs, the development of new therapeutic schemes is welcome. Nifurtimox (Nfx) and benznidazole (BZL) are classic drugs accepted for Chagas disease treatment, and both act through free radical generation. There is consensus that both drugs are effective for treating Chagas disease in its acute phase, while their therapeutic effect in the chronic phase is rather limited (Murcia et al. 2012). When performing a critical review of the literature data, the findings are controversial; it has been found that parasitological cures do not run parallel with the decrease in the serum anti-*T. cruzi* antibodies (Sguassero et al. 2015), indicating the persistence of a latent infection. What has been consistently reported is that treatment with Nfx and BZL decreases the parasite load, delaying or preventing the development of chronic chagasic cardiomyopathy (Coura & Borges-Pereira 2011), but there is no guarantee that a parasitological “cure” in chronic Chagas disease patients translates into a change in the clinical prognosis of the patient, especially when serological titres remain positive. Side effects depend on the generation of free radicals, and reactive oxygen species described for both drugs are an important issue, which causes demotivation in doctors who lack expertise in the use of both drugs. However, side effects are no longer a reason not to treat (Murcia et al. 2012).

An alternative view to developing new therapeutic drugs is the use of protocols in which the classical drugs Nfx and BZL are combined with potentially useful drugs, from the parasitological and pathophysiological point of view. The drugs to be tested should ideally have a trypanocidal effect or enhance the trypanocidal effect of Nfx or BZL, counteract the side effects of both drugs, stimulate the immune system, have an anti-inflammatory effect, and improve cardiac function; also, there must be sufficient experience in the therapeutic use of the drug in humans.

Dipyridamole (DPY) is an old drug whose mechanism of action seems to indicate that it meets the aforementioned characteristics. It has been used primarily as a platelet aggregation inhibitor in preventing cerebral thromboembolic diseases. Its mechanism of action is based on inducing elevated levels of extracellular adenosine and by inhibiting the phosphodiesterase five (PDE5) enzyme. Adenosine has cardioprotective effects by decreasing the metabolic rate due to its chronotropic, inotropic and dromotropic negative effects, and it causes coronary vasodilation, improving the coronary flow and preventing platelet aggregation, which reduces the chances of platelet thrombi development (Layland et al. 2014). Additionally, adenosine is an autacoid anti-inflammatory hormone with immunoregulatory properties that can limit the damage induced by inflammation and hypoxia (Palmer & Trevethick 2008). Furthermore, the molecular structure of DPY allows it to accept electrons, working as a free radical scavenger and antioxidant agent (Kim & Liao 2008).

Parasite persistence and immune-mediated cardiac damage are considered the main mechanism for Chagas disease progression. However, an interesting point in the pathophysiology of Chagas cardiomyopathy involves microvasculature disorders with increased platelet aggregation, resulting in vasospasm and thrombus formation and causing ischaemia and necrosis, which progressively leads to cardiac remodelling and chronic cardiomyopathy (Rossi et al. 2010). In addition, intense inflammation and free radical generation are key factors in the residual damage caused by *T. cruzi* (Vyatkina et al. 2004).

There is a need for new drugs because of a lack of efficacy of the current trypanocidal drugs to change the natural history of chronic Chagas disease cases. Therefore, given the pharmacological and therapeutic characteristics of DPY, it could help treat Chagas disease as an adjuvant drug by acting as a cardioprotective drug that should counteract some pathophysiological phenomena induced by infection and side effects induced by Nfx, decreasing the disease sequelae.

In the present paper, we study the therapeutic effect of DPY (30 mg/kg) combined with Nfx (10 or 40 mg/kg) in NMRI male mice with acute chagasic myocarditis that were treated from the third week post-infection.

## MATERIALS AND METHODS

*T. cruzi axenic cultures* - The strain of *T. cruzi* used in this work was M/HOM/VE/92/2-92-YBM. The strain has been maintained through successive passages from the vector to a susceptible host. The vectors were *Rhodnius prolixus* stage III nymphs, and NMRI mice were used as a susceptible host. Drug inhibitory effects were tested at concentrations between 1 µM and 1 mM in a final volume of 10 mL of liver infusion tryptose (LIT) medium in 50-mL Falcon tubes in triplicate. Parasitic proliferation began with an OD of 0.2 (4 x 10^6^ parasites/mL) when parasites were in a logarithmic phase of growth. Epimastigotes were cultured under continuous stirring in a rotary incubator at 28ºC for 48 to 72 h.

*In vivo model* - Animals used in this study were NMRI albino mice, adult males, 30-40 g weight, from the animal facilities of Universidad Centroccidental Lisandro Alvarado, Venezuela. Mice were kept in 40 x 25 x 15 cm stainless steel cages, with 5-10 animals per cage, at a temperature between 25-30ºC, 12-h light/darkness cycles and relative humidity of 65%. Feeding was based on concentrated feed (Perrarina®, Protinal, Venezuela), and sterilised water was provided ad libitum.

The tested drugs were as follows. (i) Nifurtimox, Lampit® (Bayer Corporation Bonima SA, El Salvador), which was packaged in 120 mg tablets. For *in vitro* experiments, tablets were pulverised in a mortar and suspended in dimethyl sulphoxide (DMSO) in a final volume (VF) of 10 mL; they were extracted under continuous stirring for 24 h and then centrifuged at 2000 rpm for 30 min. The supernatant with drug dissolved at a concentration of 41.77 mM was obtained, and the drug was tested at final concentrations between 0.1-100 µM. For *in vivo* experiments, one or four tablets were crushed and suspended in 12 mL of 1% carboxymethylcellulose, resulting in concentrations of 10 or 40 mg/mL, respectively. The dose was 10 or 40 mg/kg weight and orally administered using a micropipette. (ii) Dipyridamole (Sigma-Aldrich) stock solution of 1x10^-2^ M in absolute ethanol was prepared for *in vitro* experiments, and the drug was tested at concentrations between 0.01-1 mM. For *in vivo* experiments, suspensions of 30 mg/mL were prepared in 1% carboxymethylcellulose, and the administered dose was 30 mg/kg weight, which was orally administered with a micropipette.

The therapeutic protocol consisted of nine groups of 20 NMRI adult male mice each, including three groups of healthy mice receiving vehicle, Nfx 40 mg/kg or DPY 30 mg/kg and six groups of *T. cruzi*-infected mice treated with vehicle, Nfx 10 or 40 mg/kg, DPY 30 mg/kg, Nfx 10 mg/kg plus DPY 30 mg/kg and Nfx 40 mg/kg plus DPY 30 mg/kg.

Infected mice were orally treated daily with selected drugs until the parasitaemia was demonstrated as negative for three consecutive weeks of observation. Then, monthly parasitaemia monitoring was performed, and treatment was resumed in mice with reactivation of parasitaemia. Treatment protocols were performed for a maximum period of 6 months. Additionally, experiments were interrupted if the group mortality reached 50%.

*Electrocardiographic (ECG) studies* - These studies were conducted at the end of the treatment protocol. Under general anaesthesia with pentobarbital 50 mg/kg and xylazine 1 mg/kg ip, mice were placed in the supine position and electrodes were placed into the subcutaneous tissue. We worked with four ECG leads: DI, DII, DIII and AVF. Recording was performed in a bipolar configuration, and analogue signal was amplified with a bioamplifier (BIO Amp - ADInstruments) and transformed into a digital signal by an interface Power Lab/8sp (ADInstruments, USA). The signal generated was filtered at 60 Hz and captured at 10 kHz. The electrocardiographic signal was displayed during the experiment, stored and analysed using the LabChart program.

*Behavioural assessment* - Since it has been reported that the most serious side effects resulting from the administration of Nfx are peripheral neuropathies and central nervous system disorders of the higher functions, we decided to evaluate nociception by the hot plate and formalin tests, while motor function was evaluated by assessing motility on an open field.

The hot plate test consisted of individually placing each mouse in a box with dimensions of 32x32x26 cm. The walls were constructed of transparent acrylic and thermally conductive granite floor. On the pre-trial day, the animal was placed in the behavioural box for 10 min with the floor at room temperature. On the trial day, the animal was placed in the behavioural box with the conductive floor at 40ºC and the latency time it takes the mouse to lick his hind legs is measured up to a maximum of 5 min.

The formalin test consisted of injecting 50 µL of 5% formalin into the subcutaneous tissue in the dorsal surface of the paw. Then, each mouse was individually placed in a glass cylinder with a diameter of 30 cm and height of 30 cm at room temperature; the number of licking events in the injected paw was quantified in 5-min periods for a total of 30 min. The experiment requires a prior conditioning period a day before the measurement, placing the animal in the glass cylinder for 10 min at room temperature.

*Biochemical tests* - Creatine kinase MB (CKMB) and glutamic oxaloacetic transaminase (SGOT) plasma levels were determined using commercial kits according to the manufacturer’s instructions. Blood samples were taken by cardiac puncture using heparinised syringes. Samples were centrifuged at 2500 rpm for 5 min, and plasma was aspirated and stored in small aliquots at -70ºC until their use.

*Histopathological studies* - Mice were euthanised by exsanguination with cardiac puncture under general anaesthesia. Autopsy was performed, and heart tissue samples were fixed in 10% formalin in PBS, embedded in paraffin, cut into 200-micron slices and stained with haematoxylin-eosin or Masson trichrome.

*Data analysis* - The obtained data were expressed in absolute, percentage or average values ± standard deviation (SD; for tables) or standard error (SEM; for figures). Normality was determined with the D’Agostino Pearson omnibuss normality test. The analysis of the observed difference between two groups was performed using Student’s t test and that between more than three groups was performed using ANOVA followed by Bonferroni’s post-test. In all cases, a value of p < 0.05 was accepted as significant.

To get an IC_50_, the results obtained in the inhibition curves were analysed by nonlinear regression using the inhibition dose-response curves type based on a sigmoid equation. All analyses were performed using GraphPad Prism (San Diego, CA).

For ECG analysis, 54 healthy mice and 111 mice with acute Chagas ECG traces were included. These mice belong to the database of our laboratory raised from other studies; these mice were handled similarly as mice from the experimental protocol. ECG analysis was systematised to detect rhythm, conduction and repolarisation disorders. For quantitative disorders, we based the values of the 10th and 90th percentiles (p10 and p90) of the ECG parameters calculated in the healthy mice, accepting abnormal values as those below and above these percentiles. Also, the ECG traces were tracked for morphologic qualitative disturbances.

For rhythm disorders, we defined bradycardia and tachycardia based on lower than p10 and higher than p90 values of the heart rate, respectively. Extrasystole was defined as a premature QRS complex followed by a compensatory pause. The origins of the quantitative disorders and extrasystoles were classified as follows: sinus when the QRS complex was preceded by a P wave, nodal when there was not a P wave and the QRS complex morphology was similar to the basal traces, and ventricular when there was no P wave and the QRS morphology was different from the basal traces. Atrial fibrillation was defined based on the absence of a P wave or atrioventricular dissociation, and both had variable RR intervals.

Conduction disorders were defined as lengthening of the PR segment or QRS complex, and the period’s durations were corrected by the Bazett method. Sinoatrial conduction disorders were classified in I, II and III degree blockages. Those of I degree involved PR lengthening with values greater than p90 of healthy animals. Degree II was defined when PR intervals remain constant; however, sometimes there was no QRS complex following P waves. Degree III blockage (atrioventricular dissociation) was defined as P waves not synchronised with QRS complexes with different PP and RR interval values, while intraventricular conduction disorders were defined based on the p10 and p90 QRS complex duration.

Repolarisation disorders were defined as T wave disorders with the T wave being the positive deflection following the S wave above the isoelectric line. Quantitative T wave disorders were defined by the T wave amplitude and QT intervals (which start at the Q wave of the QRS complex and end when the T wave returns to the isoelectric line) taking p90 and p10 into account. The T wave decrease was analysed by means of an exponential decay equation with two components, defining the values of the t_1_ and t_2_ constants with their respective percentage in the amplitude of the decay. Qualitative disorders were visualised as flattening or inversion of the T wave or as post-depolarisation visualised as a wave (U wave) that emerged during the second component of the T wave decay. All quantitative analyses were calculated using LabChart data analysis software (ADInstruments).

*Ethics* - The project was approved on January 24, 2014, by the Ethics Committee of the Health Sciences School, Universidad Centroccidental Lisandro Alvarado, Barquisimeto, Venezuela. Murine manipulation was performed according to the ethical criteria for the use of experimental animals of the National Endowment for Science Technology and Innovation (http://www.cdc.fonacit.gob.ve/boletin/libro2_311003.html).

## RESULTS

**Fig. 1:**
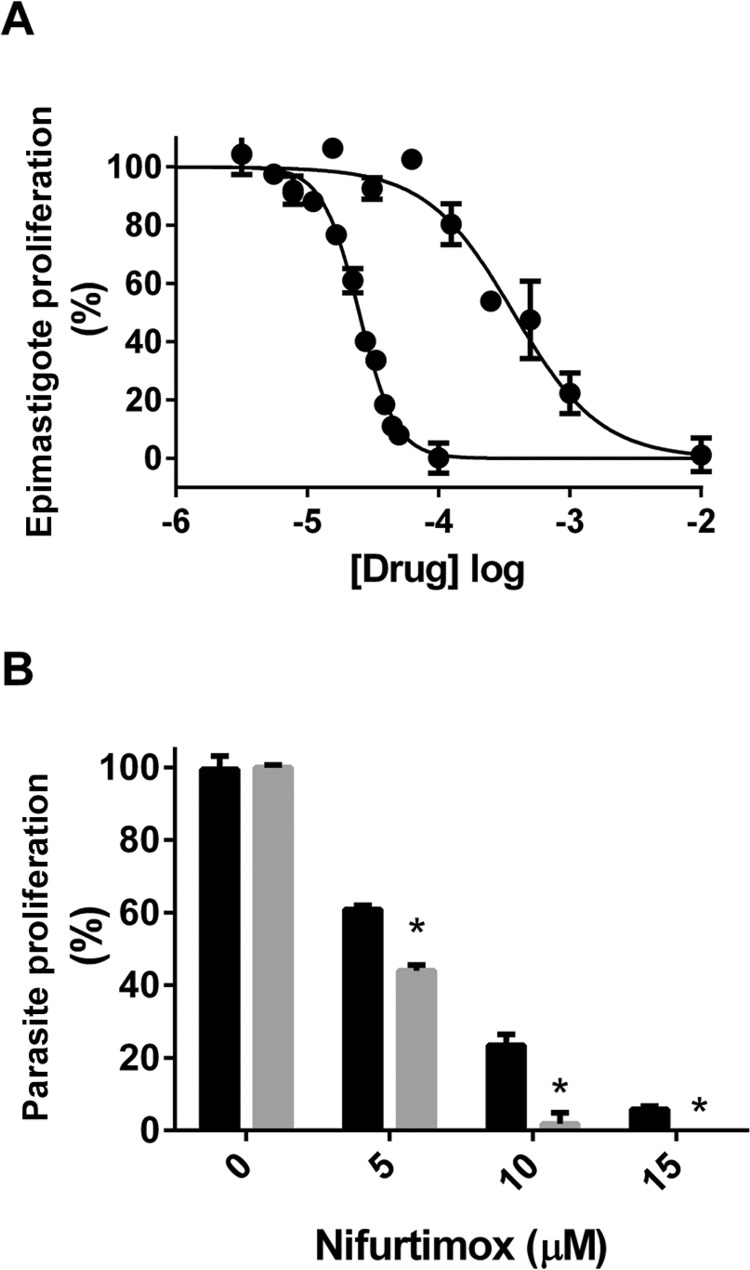
: effect of nifurtimox (Nfx) and dipyridamole (DPY) in epimastigote axenic culture. After epimastigote growth in liver infusion tryptose (LIT) medium at 28ºC under continuous rotational shaking until they achieved logarithmic growth with a density of 4 x 106 parasites/mL, moment Nfx (1 µM to 1 mM) and/or DPY (10 µM to 1 mM) was added and then incubated for 48 h. (A) Normalised dose response curves for Nfx (left) and DPY (right); observe that Nfx had 10 times more potency than DPY on the inhibition of epimastigote proliferation. (B) DPY (20 µM) was assayed at 5, 10 and 15 µM doses of Nfx; observe that DPY potentiated the effect of Nfx.

*Nfx and DPY potency in vitro* - Inhibition curves performed on epimastigotes in the logarithmic phase of growth gave IC_50_ values of 21.53 ± 2.13 and 372 ± 52 µM for Nfx and DPY, respectively (Fig. 1A). To study the NFX/DPY interaction, we determined the effect of 20 µM DPY on the inhibition induced by 5, 10 and 20 µM NFX. Fig. 1B clearly shows that DPY enhances the inhibitory effect of Nfx on parasite proliferation.

**Fig. 2:**
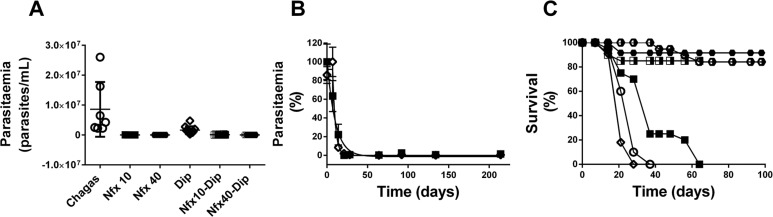
: parasitaemia and survival in infected mice treated with nifurtimox (Nfx) and/or dipyridamole (DPY). Parasitaemia was quantified using Newbauer’s camera seeded with 5 µL of blood diluted 1:10 in NaCl 0.9%; mortality was registered until 50% of mice for the determined group died. (A) Shows parasitaemia obtained at the end of the experiment; observe that groups treated with Nfx alone or combined with DPY had parasitaemia close to 0, while parasitaemia persisted in control Chagas mice and DPY-treated mice. However, parasitaemia with DPY was significantly lower than control Chagas mice. (B) Shows parasitaemia evolution as a function of time in mice treated with Nfx 40 mg/kg (black filled squares) and Nfx 40 mg/kg plus DPY 30 mg/kg (open diamond); parasitaemia achieved values close to 0 from the 25th day, and the DPY-Nfx group tended to increase parasitaemia in the first week. (C) Shows survival curves for control Chagas (open circle), DPY 30 mg/kg (open diamond), Nfx 10 mg/kg (black filled square), Nfx 40 mg/kg (hexagon total black filled), Nfx 10 mg/kg plus DPY (square half black filled) and Nfx 40 mg/kg plus dipyridamole (hexagon half black filled) groups. The control Chagas and DPY and Nfx 10 mg/kg groups achieved 100% mortality and mortality between 10 and 20% in the reamaining groups, respectively.

*Parasitaemia* - Parasitaemia values obtained at the end of the protocol in the Nfx-treated groups compared with the control Chagas group are shown in Fig. 2, panel A. All treated groups, including the DPY group, showed significantly lower parasitaemia compared to the Chagas control group. In panel B, the evolution of parasitaemia is displayed in relation to the time course of treatment with Nfx to 40 mg/kg, alone and combined with DPY, obtaining t_50_ values (days needed to decrease parasitaemia by 50%) of 6.9 and 9.3 days, respectively. In panel C, the percentages of survival are shown, demonstrating that the control Chagas, DPY and Nfx 10 mg/kg groups showed 100% mortality, while mice treated with Nfx 40 mg/kg, Nfx 10 mg/kg or 40 mg/kg associated with DPY showed survival percentages of 92, 84 and 85%, respectively.

*ECG* - To visualise clearly ECG changes induced by treatment, we first analysed ECG traces in 74 healthy mice and 131 infected mice in the acute phase of Chagas disease and then compared them with the traces obtained in mice belonging to the experimental groups.

In chagasic mice, compared with healthy mice, ECG disorders in virtually all parameters were observed, except for the S wave amplitude, P wave axis, second component of the T wave decay (t_2_) and in the QT interval length. Healthy mice displayed very few ECG qualitative disturbances; we only observed sinus extrasystole in 6 (8.1%) individuals. By contrast, 65.4% of chagasic mice displayed some type of disturbance, which was most frequently a post-depolarisation (U) wave (65.4%), sinus extrasystole (23.5%), ventricular extrasystole (21.3%), two independent P waves (19.8%) and a bifid P wave (17.6%). Also, we observed severe rhythm disorders as non-sustained supraventricular (10.2%) or ventricular tachycardia (1.4%), atrial fibrillation (4.6%) and atrioventricular dissociation (6.2%) (Tables I, II, III).

**TABLE I:** Electrocardiographic (ECG) parameters found in untreated and dipyridamole (DPY) or nifurtimox (Nfx) treated healthy NMRI mice

ECG/Healthy	Untreated			DPY		Nfx	
		
	Mean ± SD	Percentile		Mean ± SD	p value	Mean ± SD	p value

		10%	90%				
HR	333.8 ± 103.1	216.6	453.5	252.8 ± 21.9	↓ 0.04	344.7 ± 112.6	0.80
PR	39.7 ± 7	34.9	52.9	43.4 ± 4.7	0.67	44.1 ± 14.6	0.59
PR Bazett	96.2 ± 14.1	80	108	89.1 ± 9.7	0.19	103.3 ± 23.5	0.24
QRS	11.2 ± 1.8	9	14.1	13.2 ± 1.1	↑ 0.00	12.3 ± 2.15	0.18
QRS Bazett	25.7 ± 3.6	21.5	30.5	27.2 ± 2.5	0.26	29.3 ± 4.5	↑ 0.02
QT	79.7 ± 42.5	25.9	141.4	75.5 ± 29.4	0.79	80.4 ± 70.2	0.97
QTc	176.8 ± 79.2	68.4	291.9	155.6 ± 63.7	0.49	182.5 ±136.7	0.86
Pamp	89.3 ± 42.9	43.7	143.3	74.7 ± 39.4	0.38	86.9 ± 70	0.89
Ramp	1140 ± 312.2	740.5	1548	855.4 ±153.5	↓ 0.01	1069 ± 282.1	0.58
Samp	214 ± 189.1	18.4	411.9	267 ± 161.4	0.47	290.3 ±241.2	0.34
Tamp	409.7 ± 125.6	255.4	589.1	395.2 ± 74.1	0.76	387.4 ± 75.8	0.66
P axis	47.7 ± 26.5	8	80.5	40.2 ± 38.1	0.44	30.6 ± 18.6	0.13
QRS axis	83.1 ± 9.3	75	89	91.5 ± 5.1	↑ 0.02	77.8 ± 14.5	0.19
T axis	85.9 ± 3.4	81	89	89.4 ± 4.8	↑ 0.01	85.5 ± 7.1	0.77
t_1_	4.1 ± 0.9	2.8	5	4.4 ± 0.8	0.39	3.7 ± 1.6	0.40
t_1%_	90.5 ± 9.7	75.8	100	89.4 ± 6.9	0.76	84.6 ± 22.8	0.19
t_2_	67.3 ± 43.8	38.4	113.3	90.4 ± 76.3	0.14	31.2 ± 48.3	↓ 0.01
t_2%_	9.4 ± 9.7	0	24.1	10.6 ± 3.8	0.87	15.3 ± 22.8	0.26
Tad 5ms	34.2 ± 7.4	23.9	43	34.6 ± 5.5	0.94	34.7 ± 11.2	0.92
Tad 10 ms	18.3 ± 6.4	8.9	26.5	18.4 ± 4.4	0.85	18.9 ± 15.1	0.98
Tad 20ms	11.5 ± 6.2	2.6	18.1	10.8 ± 4.5	0.69	12.4 ± 16.8	0.87
Tad 40ms	8.1 ± 5.5	1	14.9	8.6 ± 4.5	0.99	13.7 ± 7.9	0.09
Tad 60ms	6 ± 5.3	0	11.8	6.8 ± 1.3	0.09	9.1 ± 4	0.40

t: T wave decay constant; Tad: T amplitude decay at each indicated time; arrows down or up indicate a decrease or increase of the ECG parameters. P values shown are related to values obtained in untreated mice. Doses were 30 and 10 mg/Kg of DPY and Nfx, respectively.

**TABLE II:** Electrocardiographic (ECG) parameters of acute Chagas NMRI mice treated with dipyridamole (DPY), nifurtimox (Nfx) alone or combined with DPY, compared to untreated control group

ECG Parameters	Untreated			DPY			Nfx			DPY + Nfx		
			
	Mean ± SD	Percentile		Mean ± SD	Analysis		Mean ± SD	Analysis		Mean ± SD	Analysis	
			
		10%	90%		Healthy	Chagas		Healthy	Chagas		Healthy	Chagas
HR	↑ 382.9 ± 86.2	279.1	503.6	405.6 ± 32.6	↑ 0.03	0.75	419 ± 100.5	↑ 0.00	0.36	377.3 ± 89.6	0.07	0.96
PR	↑ 54.8 ± 12.5	40.2	70.5	56.1 ± 10.9	↑ 0.00	0.94	55.5 ± 10.7	↑ 0.00	0.86	55.8 ± 12.6	↑ 0.00	0.93
PR Baz	↑ 137.9 ± 27.4	106.3	177.4	144.3 ± 25.7	↑ 0.00	0.69	146.2 ± 18.11	↑ 0.00	0.42	136.9 ± 25.5	↑ 0.00	0.99
QRS	↑ 13.9 ± 2.9	10.3	17.4	11.4 ± 1.2	0.98	↓ 0.03	12.2 ± 2.4	0.99	↓ 0.00	11.4 ± 1.5	0.96	↓ 0.01
QRS Baz	↑ 32.4 ± 9.8	15.1	45.2	32.5 ± 8.4	0.06	0.99	32 ± 10.6	↑ 0.03	0.96	28.2 ± 3.6	0.64	0.08
QT	90.1 ± 40.4	37.2	141	75.9 ± 23.4	0.96	0.60	74.5 ± 32.2	0.91	0.41	81.1 ± 34.6	0.99	0.68
QTc	↑ 215.6 ± 84.5	85.3	319.6	198 ± 55.4	0.76	0.82	187.1 ± 68.5	0.91	0.48	194.9 ± 68	0.70	0.60
P amp	↓ 47.3 ± 30.5	10.2	82.9	55.1 ± 19.8	↓ 0.01	0.76	75 ± 27.6	0.24	↑ 0.01	78.6 ± 27.5	0.52	↑ 0.00
R amp	↓ 664.3 ± 311.5	270.6	1033	861.2 ± 453.9	↓ 0.04	0.15	783.5 ± 333.7	↓ 0.01	0.34	956.1 ± 161.4	0.08	↑ 0.00
S amp	265.8 ± 190.5	34.9	564.7	276.6 ± 230.8	0.51	0.98	206.7 ± 162.8	0.99	0.52	286.2 ± 227.6	0.21	0.90
T amp	↓ 129.6 ± 191	95.2	398	184.9 ± 131.1	↓ 0.01	0.81	326.1 ± 234.8	0.23	↑ 0.00	441.3 ± 124.4	0.73	↑ 0.00
P axis	54.3 ± 44.3	15.1	94.6	51.4 ± 28.3	0.97	0.98	79.7 ± 6.5	↑ 0.01	0.06	83.6 ± 8.13	↑ 0.00	↑ 0.01
QRS axis	↓ 71.3 ± 43.2	20.8	95.6	76.1 ± 7.9	0.86	0.88	77.6 ± 15	0.87	0.75	81.8 ± 8.78	0.99	0.39
T axis	↓ 58.7 ± 58.2	77.1	93.9	76.5 ± 9.3	0.85	0.57	81.5 ± 11.2	0.94	0.24	85.6 ± 5.6	0.99	0.05
t_1_	↑ 7.6 ± 3.8	3.5	14.3	7.9 ± 3	↑ 0.00	0.74	4.7 ± 1.2	0.70	↓ 0.01	4.6 ± 0.9	0.75	↓ 0.01
t_1%_	↓ 55.2 ± 31.7	8.2	100	89 ± 16.46	0.98	↑ 0.00	79.7 ± 24.5	0.38	↑ 0.01	81.4 ± 21.6	0.36	↑ 0.01
t_2_	60.3 ± 44.9	13.4	124.8	144.2 ± 152.9	↑ 0.01	↑ 0.00	72.6 ± 32.6	0.95	0.76	61.1 ± 23.6	0.92	0.99
t_2%_	↑ 48.1 ± 28.5	8.5	91.1	29.3 ± 11.8	0.30	0.34	37.1 ± 21.4	↑ 0.01	0.48	35 ± 17.1	↑ 0.00	0.22
Tad 5ms	↑ 71.6 ± 12.8	51.9	86.8	62.7 ± 13.4	↑ 0.00	0.07	48.1 ± 18.9	↑ 0.00	↓ 0.00	47.9 ± 12.6	↑ 0.00	↓ 0.00
Tad10 ms	↑ 48.5 ± 15.7	24.6	70.3	35.4 ± 13.6	↑ 0.01	0.05	27.3 ± 16.7	0.12	↓ 0.00	28.5 ± 13.4	↑ 0.01	↓ 0.00
Tad 20ms	↑ 33.2 ± 17.5	8.2	59.7	23.4 ± 14.6	0.05	0.17	21.9 ± 14.1	↑ 0.04	↓ 0.04	19.7 ± 11.2	0.05	↓ 0.00
Tad 40ms	↑ 27.6 ± 16.2	4.4	49.1	17.7 ± 10.6	0.17	0.14	13.9 ± 11.6	0.42	↓ 0.00	15.2 ± 10.12	0.14	↓ 0.00
Tad 60ms	↑ 16.8 ± 12	1.2	31.9	8.5 ± 6	0.69	0.11	3.8 ± 6.3	0.82	↓ 0.00	10.3 ± 7.5	0.11	0.05

Baz: Bazzet; t: T wave decay constant; Tad: T wave amplitude decay at each indicated time; arrows down or up indicate a decrease or increase of the ECG parameters. P values shown are related to values obtained in untreated mice. Nfx and DPY doses were 10 and 30 mg/Kg respectively.

**TABLE III:** Electrocardiographic (ECG) rhythm, conduction and repolarisation qualitative disorders

Heart rhythm disorders	Group									

	Healthy		Chagasic		Nifurtimox (Nfx)		Dipyridamole (DPY)		DPY + Nfx	
				
	n	(%)	n	(%)	n	(%)	n	(%)	n	(%)
Sinus extrasystole	6	8.1	32	23.5	2	25	2	25	0	0
Ventricular extrasystole	0	0	29	21.3	3	37.5	0	0	1	12.5
Nodal extrasystole	0	0	4	2.9	1	12.5	1	12.5	0	0
Supraventricular tachycardia	0	0	14	10.2	0	0	0	0	0	0
Ventricular tachycardia	0	0	2	1.4	2	25	0	0	0	0
Atrial ectopics beats	0	0	8	6.2	0	0	0	0	0	0
Atrioventricular dissociation	0	0	8	6.2	0	0	0	0	0	0
Atrial fibrillation	0	0	6	4.6	0	0	0	0	0	0
Second-degree AV block	0	0	3	2.3	0	0	0	0	0	0
Two P waves	0	0	27	19.8	7	87.5	3	37.5	5	62.5
Bifid P wave	0	0	24	17.6	0	0	0	0	1	12.5
Post-depolarisation (U) wave	0	0	89	65.4	4	50	2	25	3	37.5
Flattened T wave	0	0	12	8.8	3	37.5	0	0	0	0

Total n were 74 and 136 for healthy and chagasic mice respectively; for treated groups were eight each one. Nfx and DPY doses were 10 and 30 mg/Kg respectively.

In Nfx-treated healthy mice, an increase in the corrected QRS length and decrease in the value of the second component of the T wave decay (t_2_) were observed. In healthy mice treated with DPY, a decrease in the heart rate and R wave amplitude and an increase in the QRS length and in QRS and T axes values was obtained. No ECG qualitative disturbances were induced with both drugs.

**Fig. 4:**
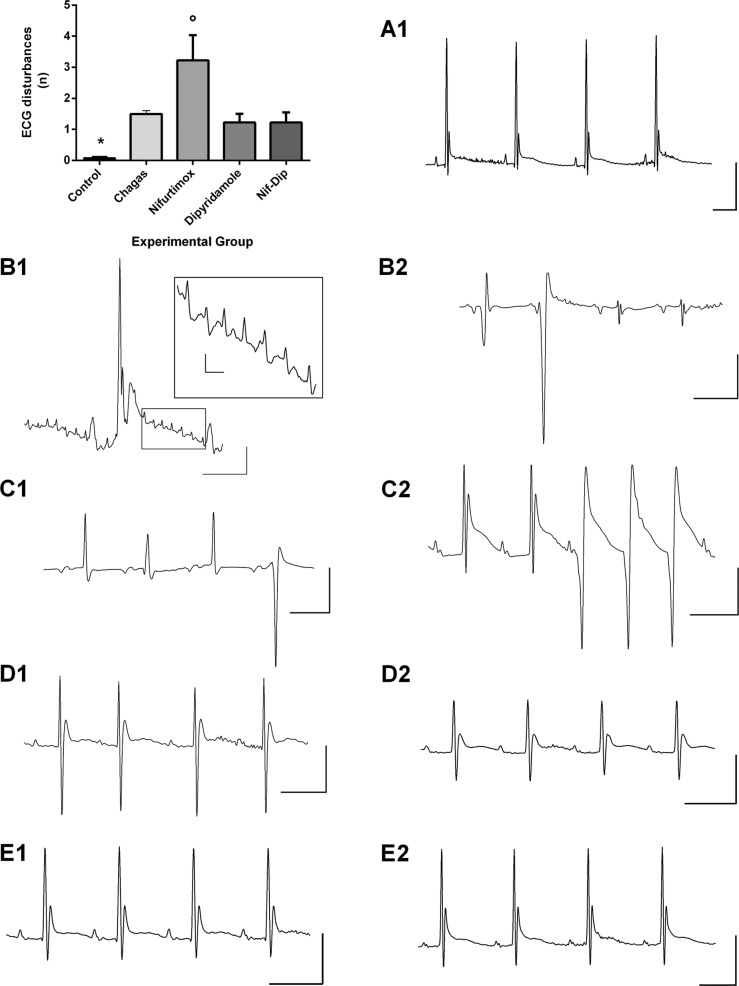
: typical electrocardiographic (ECG) traces and qualitative ECG disorders observed in healthy, untreated and treated chagasic mice. The mean ± standard error (SEM) of the qualitative ECG disorders (rhythm, conduction and repolarisation disorders) observed in mice of the different experimental groups are shown in the upper panel on the left. Healthy mice have practically no disorders, whereas the group of chagasic mice treated with nifurtimox (Nfx) had a significantly greater increase in these disorders compared with the remaining groups. In panel A1, we show a typical record of a healthy mouse. Traces of mice with chagasic myocarditis are shown in panels B1 and B2; in B1, we can observe low-voltage QRS complexes with a Rr’ wave suggestive of His intranodal block and multiple rhythmical atrial ectopics that are magnified in the inserted box suggestive of atrial flutter; in B2, we can perceive a complex rhythm disorder with ventricular extrasystoles, a very low voltage QRS complex, a deep S wave and a negative T wave. Traces of mice with chagasic myocarditis treated with Nfx at doses of 10 mg/kg of body weight are shown in panels C1 and C2 where there is a worsening in the qualitative disorders. In C1, there is atrioventricular dissociation, a negative-positive biphasic P wave, and diminished voltage QRS complex with variable morphology, including ventricular extrasystole and negative T wave. In C2, there is a double P wave and peaked T wave with an evident delay in its decay, which delineates a post depolarisation phenomenon (U wave) and no sustained ventricular tachycardia. In D1 and D2, traces from mice with chagasic myocarditis treated with dipyridamole are shown with clear improvement in the qualitative aspects of the electrocardiogram; however, repolarisation disorders and a decrease in the voltage of the QRS complex persist (D2). Traces of mice with chagasic myocarditis treated with Nfx 10 mg/kg plus dipyridamole are shown in E1 and E2, where there are practically no ECG disorders (compare with trace A1). *: indicates p < 0.05 when comparing the control with the other experimental groups, º: it indicates p < 0.05 when comparing the group treated with Nfx compared to the other experimental groups. The vertical bars are equivalent to 500 μV and horizontal bars are 100 ms for all traces, by except of panel B1 where vertical and horizontal bars are 150 µV or ms, respectively; also in the insert vertical bar is equivalent to 50 µV and the horizontal bar is to 25 ms.

In mice with acute Chagas disease treated with DPY, the same ECG disorders as observed in the Chagas control group were seen; however, DPY significantly shortened the QRS length compared with chagasic mice (Table II). Notwithstanding, ECG traces in these mice were improved and they were close to ECG traces from chagasic mice treated with Nfx 10 mg/kg plus DPY (Fig. 4). Moreover, the ECG qualitative disturbances were lower compared with the Nfx 10 mg/kg treated group (Fig. 4, Table III).

Chagas disease Nfx-treated mice had improved atrial depolarisation and ventricular repolarisation disorders; however, a significant increase in the T amplitude decay values at 5 and 20 ms persisted compared with control mice. Although the QRS length value was significantly reduced in the treated group compared to chagasic mice, the corrected QRS length value was similar to that observed in chagasic mice and significantly higher than in healthy mice, indicating that intraventricular conduction disorders persisted in treated mice (Table II). Noticeably, in these mice, there were increases in ECG qualitative disorders compared with all groups (Table III), especially severe rhythm disorders, such as non-sustained ventricular tachycardia and multiples ventricular ectopics, as well as ischaemic disorders such as flattened or inverted T wave and post-depolarisation wave (Fig. 4, Table III).

In mice with acute Chagas disease treated with Nfx 10 mg/kg combined with DPY, an improvement in most of the analysed parameters was observed. However, a persistence of the prolonged PR interval was observed and ventricular repolarisation improvement was partial because an increase in the T wave decay amplitudes at 5 and 10 msec persisted compared to values obtained in healthy mice (Table II). As expected, ECG traces were similar to healthy mice; however, ECG qualitative disturbances were higher in the Chagas mice (Fig. 4, Table III).

*Histopathology* - The hearts from the groups treated with Nfx 10 or 40 mg/kg, alone or in combination with DPY, had a significant decrease in their weights compared with the Chagas control group. Spleens from groups treated with Nfx 40 mg/kg alone, Nfx 10 mg/kg plus DPY and Nfx 40 mg/kg plus DPY had a significant decrease in the weight compared with the control Chagas group. In mice treated with Nfx 40 mg/kg, a significant decrease in liver weight was also observed (Table IV).

**TABLE IV:** Biochemistry and morphohistopathological parameters in NMRI mice treated or not with nifurtimox (Nfx) and/or dypiridamol (DPY)

Experimental group	Biochemistry		Autopsy				Histopathology		

			Organ Weight						
		
	AAT (U/L)	CKMB (U/L)	Body (gr)	Heart (mg/gr)	Spleen (mg/gr)	Liver (mg/gr)	Inflammation (grade)	Parasite nest n°/field	Fibrosis (%)
Control Chagas	348,0 ± 61.7	65.4 ± 6.0	41.8 ± 1.9	7.3 ± 0.8	22.5 ± 2.7	62.6 ± 3.8	5.3 ± 0.1	2.9 ± 0.1	63.9 ± 19.5
Nfx 10	276.3 ± 44.4	24.2 ± 6*	42.1 ± 1.4	5.1 ± 0.2*	19.1 ± 2.2	59.8 ± 3.0	2.7 ± 0.5*	0.8 ± 0.2*	36.1 ± 5.5*
Nfx 40	82.4 ± 9.3*	22.1 ± 4.7*	49.9 ± 2.3*	5.0 ± 0.3*	5.84 ± 0.4*	50.7 ± 0.9*	1.6 ± 0.4*	0.0 ± 0.0*	54.2 ± 11.3
Dip	293.9 ± 18.4	55.1 ± 5.9	41.14 ± 1.8	5.9 ± 0.3*	21.8 ± 2.8	63.3 ± 3.3	5.5 ± 0.3	2.7 ± 0.2	--
Nfx 10-Dip	136.3 ± 9.6*	26.4 ± 3.5*	41.6 ± 0.8	5.5 ± 0.2*	14.6 ± 1.2*	56.8 ± 1.5	1.4 ± 0.2	0.4 ± 0.2*	27.1 ± 7.1
Nfx 40-Dip	67.0 ± 5.6*	12.2 ± 1.5*	52.6 ± 2.3*	5.9 ± 0.3*	8.54 ± 1.3*	58.3 ± 3.1	1.1 ± 0.3	0.2 ± 0.2*	40.3 ± 3.4

*: means p < 0.05 as compared with control Chagas.

**Fig. 5:**
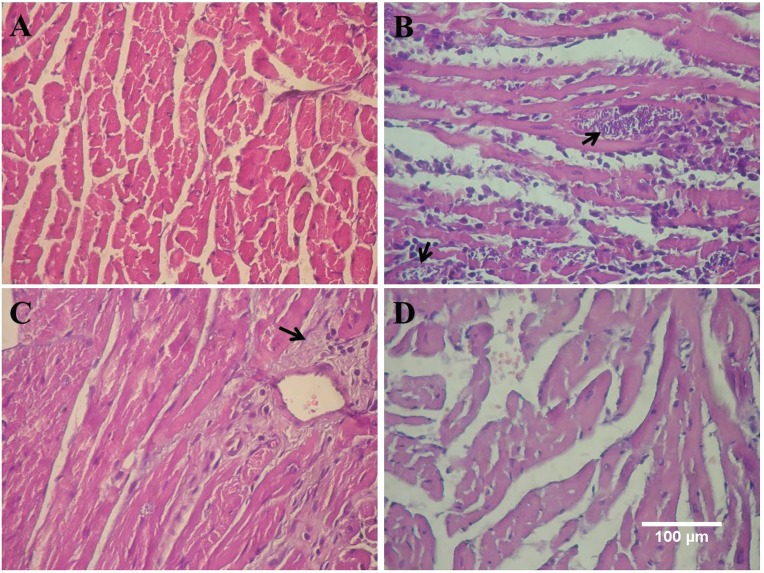
: histopathological findings in haematoxylin-eosin stained heart tissue. (A) Shows a heart tissue sample from healthy mice; (B) from *Trypanosoma cruzi*-infected mice where amastigote nests (arrows), mononuclear infiltrate and myocitolisis can be observed; (C) 40 mg/kg Nfx-treated mice lacked amastigotes present, but fibrosis areas were present; and (D) 40 mg/kg Nfx plus 30 mg/kg-treated mice lacked evident lesions or parasite forms.

In groups treated with Nfx 10 and 40 mg/kg alone or in combination with DPY, a significant decrease in inflammatory cell infiltrates was observed compared to the control group. The DPY-treated group showed an inflammatory cell infiltrate similar to the Chagas control group (Fig. 5, Table IV).

In groups treated with Nfx 10 and 40 mg/kg alone or in combination with DPY, a significant decrease in the amastigote nest number was observed, while the group treated with DPY alone showed a similar number as in the Chagas control group (Fig. 5, Table IV).

**Fig. 6:**
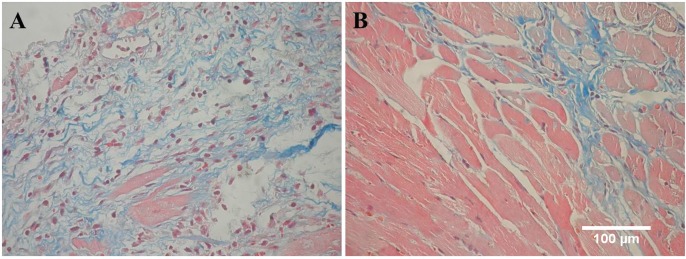
: histopathological findings in heart tissue stained by Masson’s trichrome technique. (A) Shows a sample from *Trypanosoma cruzi*-infected mice and (B) from 40 mg/kg nifurtimox (Nfx)-treated mice; note that there is extensive lax fibrosis with persistent inflammatory infiltrate and an absence of cardiomyocytes in chagasic heart sections, while in the heart sections of mice treated with Nfx, the fibrosis is less extensive, compact and interfibrilar.

Likewise, in the groups treated with Nfx 10 or 40 mg/kg associated with DPY, a significant decrease in the fibrosis was observed compared to the control Chagas group (Fig. 6, Table IV).

*Plasma enzymes* - In the Chagas control and DPY-treated groups, a significant increase in the serum CKMB activity was observed, while mice treated with Nfx alone at both doses or combined with DPY had decreased CKMB levels, achieving values similar to those in healthy mice. The SGOT serum activities were also significantly increased in the Chagas control, DPY and Nfx 10 mg/kg groups, while in mice treated with Nfx 40 mg/kg alone or in combination with DPY at both doses, the serum SGOT levels were significantly decreased and similar to healthy mice (Table IV).

**Fig. 3:**
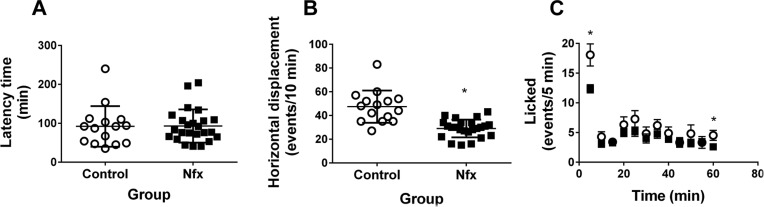
: assessment of nifurtimox (Nfx) side effects by behavioural tests in healthy mice. (A) Shows results obtained from the hot plate test, which assesses cutaneous pain sensitivity, and no significant difference was observed between healthy mice and healthy mice treated with 40 mg/kg Nfx for 30 days. (B) Shows results obtained from the open field test, which assesses horizontal and vertical motility; Nfx-treated healthy mice displayed significantly less motility compared to healthy untreated mice. (C) Shows results obtained from the formalin test, which assesses neural and inflammatory pain sensitivity; licking was only significantly higher at 5 and 45 min in Nfx-treated healthy mice; however, in the other time windows, no differences were observed.

*Behavioural effect of Nfx in healthy mice* - Mice treated with Nfx exhibit similar behaviour in the nociceptive hot plate test and formalin test late licking behaviour; however, a significant decrease in early licking behaviour during the formalin test was observed. Moreover, in the group treated with Nfx 40 mg/kg, a significant decrease in horizontal displacement movements was observed compared with healthy mice (Fig. 3).

## DISCUSSION

In the present paper, we confirm that Nfx is an efficient trypanocidal drug, although its effect depended on the dose. In our animal model, the dose of 40 mg/kg was therapeutic; parasitaemia was suppressed on day 20 post-treatment and remained negative until the end of the experiment. Also, 92% of the treated mice survived and showed no cardiomegaly, hepatomegaly or splenomegaly, and heart histopathological studies did not reveal free or nest amastigotes, while the inflammatory infiltrate was mild. By contrast, Nfx at 10 mg/kg was subtherapeutic, as revealed by the 80% mortality, continuous parasitaemia increase in the first three weeks of treatment, persistence of amastigotes in heart tissue, splenomegaly, abnormal EKG and elevated blood SGOT activity. The 40 mg/kg Nfx therapeutic dose is above the usually recommended dose of 10 mg/kg for treating Chagas disease in humans.

Despite being a drug that was discovered in the last century, there have been few systematic, comprehensive and complete studies in murine models on the therapeutic effect of Nfx. The data presented here represent one of the most comprehensive studies to date. Other studies have used high doses of the drug, obtaining conflicting results, while Bustamante et al. (2014) obtained a 95 to 100% cure rate at 100 mg/kg in a continuous or intermittent protocol applied in C57BL/6 (Ly5.2) mice and Wong-Baeza et al. (2015) obtained a slight decrease in parasitaemia with the same dose for 50 days in NIH albino mice. This discrepancy may be explained by the strains of mice used; however, the response could also depend on the assayed strain of *T. cruzi*, which is apparent from the work of Andrade et al. (1985), who found that type I strains (high and early parasitaemia with macrophage tropism) had high susceptibility (56 ± 16% cure), type II strains (high and late parasitaemia with heart tropism) had medium to high susceptibility (52 ± 11% cure), and type III strains (low parasitaemia with skeletal muscle tropism) had low susceptibility (0.45 ± 0.45% cure) to therapeutic schemes based on Nfx 200 mg/kg for four days followed by 50 mg/kg for five days/week for 90 days. Furthermore, Faúndez et al. (2008) found decreased parasitaemia at doses of 2.5 and 10 mg/kg with 25 and 100% survivals, respectively.

When comparing the effectiveness obtained in this work in cultured epimastigotes (IC_50_ = 21.53 µM) with Nfx doses used in *in vivo* experiments, where 10 and 40 mg/kg are equivalent to 34.8 and 139.23 µmol/kg, respectively, these data confirm that 10 mg/kg is a subtherapeutic dose, while 40 mg/kg is almost a therapeutic dose based on the IC_50_. However, when we analysed the drug bioavailability, which is very low (5.25%) due to rapid and extensive metabolism in the liver, where it is subjected to nitroreduction by cytochrome P-450 reductase, the serum concentration of Nfx achieved after an oral dose of 15 mg/kg (52 µmol/kg) would be 2.73 µM serum (Paulos et al. 1989), which is far below the therapeutic concentrations. Therefore, the drug potency *in vivo* is increased. Increased potency could be explained through drug metabolism during which host cells generate free radicals that facilitate parasite lysis (Letelier et al. 2004, Bartel et al. 2007).

Two points have been crucial to the reluctance to Nfx routine use: the reasoned but controversial lack of efficacy and frequent side effects (Murcia et al. 2012). In this paper, we evaluated the side effects of Nfx at a dose of 40 mg/kg in healthy mice and found slight but significant disturbances in the red blood cell count (even when diminished, it remained within normal limits for the species), decreased motility and decreased early nociceptive response to formalin; we did not observe disorders in animal weight, in the counting and distribution of white blood cells and in nociception to thermal stimuli. Therefore, our results indicate that psychic excitability and polyneuropathies with paraesthesia observed in humans are not observed in mice treated with Nfx.

In a phase I study performed in paediatric patients on the therapeutic effect of Nfx alone or in combination with cyclophosphamide and topotecan for treating refractory neuroblastoma with multiple relapses, it was determined that the maximum tolerated dose in these compromised patients was 30 mg/kg (Sholler et al. 2011), which is very close to the therapeutic dose raised in the present paper. In addition, we observed that chagasic mice treated with Nfx 40 mg/kg had an increased weight and maintained a healthy trophism in terms of the coat appearance and instinctive behaviours. By contrast, in chagasic mice treated with Nfx 10 mg/kg, alopecia, piloerection, crusted lesions and defensive muscle tone were observed (results not shown). To reconcile data reported in the literature with data reported in the present study, we propose that the side effects are a greater reflection of concurrent Chagas disease pathophysiological phenomena than the drug itself. In fact, the toxic effect of Nfx may depend on its metabolism by host cells and parasites.

Within reductions catalysed by the oxidative cytochrome P450 system in the host, the best known is the single electron reduction of nitro groups, leading to the formation of -NO2^-^, which can undergo redox recycling and generate radical superoxide anion (O2^-^) and hydroxyl radicals (HO^-^) in the presence of O_2_ and can generate oxidative stress (Letelier et al. 2004, Bartel et al. 2007). The cytochrome P450 oxidative system is strongly expressed in leukocytes; therefore, in an inflammatory process such as Chagas disease, treatment with Nfx in the presence of inflammatory cells could amplify oxidative stress and may explain the increase in ECG disturbances observed in chagasic mice treated with subtherapeutic doses. Using this dose, the inflammatory cell infiltrate continues given the persistence of the parasite in tissues and the side effects are therefore dependent on oxidative stress generated by the metabolism of Nfx in inflammatory cells. With therapeutic doses, the parasite is removed; inflammation ceases, and oxidative stress induced by Nfx metabolism in leukocytes is prevented, decreasing collateral side effects.

In this paper, we demonstrate for the first time that DPY has a trypanocidal effect *in vitro* on *T. cruzi* epimastigote proliferation and decreases parasitaemia in infected NMRI mice. However, the amastigote density in cardiac tissue was not affected by the drug. The effect of DPY on epimastigote proliferation has previously been reported in *T. brucei*, where an IC_50_ of 30 µM was obtained (de Koning et al. 2012). The dose of DPY used in the *in vivo* model was subtripanocidal because 30 mg/kg equals 60 µM. Moreover, assuming a bioavailability between 37 and 66%, we would reach serum concentrations between 22.2 and 39.6 µM, respectively, which is 9.39 to 16.75 times lower than the IC_50_ *in vitro*. At these doses, between 5 and 15% of the maximum trypanocidal effect would be obtained, which is in perfect agreement with the results obtained *in vivo* with the use of DPY alone. The lack of effect on tissue amastigotes may reflect that these parasitic forms do not depend on the extracellular adenosine as a source of endogenous purines; therefore, they are not sensitive to DPY (Guimarães & Gutteridge 1987).

Notwithstanding the trypanocidal action of DPY, its functional effect should be considered; like other authors, we think that the cardiovascular benefits of this drug have been undervalued, and the beneficial effect observed in this study could be related to its mechanism of action. First, DPY induces coronary vasodilation mediated by nitric oxide and inhibits platelet aggregation mediated by PGI2. This effect results from PDE5 enzyme inhibition, causing elevation of the intracellular cAMP and cGMP levels (Kim & Liao 2008). Moreover, by blocking nucleotide transporters, DPY elevates extracellular adenosine levels, which act on A2 receptors and potentiate intracellular elevation of cAMP levels (Layland et al. 2014). The consequence of this mode of action is improved coronary irrigation and prevention against new platelet and hematic thrombi formation, counteracting pathophysiological phenomena related to diffuse ischaemia in the coronary microvasculature. Moreover, it has been reported that DPY improves cardiac contractility of hypokinetic areas and ejection fraction in patients with Chagas cardiomyopathy (Kuschnir et al. 1983).

Second, DPY is cardioprotective, an effect mediated by adenosine acting on A1 and A3 receptors, which would optimize cardiac work according to the availability of energy sources, inhibit early apoptotic phenomena and prevent the occurrence of lethal arrhythmias (Layland et al. 2014). In this sense, it has been reported that endogenous adenosine acting on A1 receptors generates negative chronotropic and dromotropic effects, reducing the incidence of ventricular arrhythmias induced by ischaemia-reperfusion in isolated rat hearts (Lee et al. 1994).

Third, DPY is an immunomodulator drug, which is mainly mediated by A2A receptors. Adenosine acting on A1 and A3 receptors promotes the recruitment of immature dendritic cells to sites of inflammation, while it promotes the differentiation of T cells to an anti-inflammatory TH2 profile via A2A receptors. This scenario promotes decreased levels of IL6 and TNFa, while the IL10 levels are increased. This balance promotes an anti-inflammatory state in heart tissue that counteracts necrotic phenomena, fibrosis and cardiac remodelling associated with severe inflammation (Haskó et al. 2008). Elevated levels of TNF-α and IL-6 have been associated with advanced stages of Chagas disease and negatively with the cardiac ejection fraction (Mendoza et al. 2005, López et al. 2006), while high levels of IL-10 have been observed in asymptomatic patients in the indeterminate phase of Chagas disease, and patients who have developed cardiac disorders have reduced levels of this cytokine (Sousa et al. 2014).

Finally, the molecular structure of DPY allows it to accept electrons, acting as a free radical scavenger, whose capacity is even greater than α-tocopherol and vitamin C (Kim & Liao 2008). During Chagas cardiomyopathy development, the myocardium is continuously exposed to injury caused by the release of free radical products of mitochondrial damage (Vyatkina et al. 2004); therefore, the beneficial effect of DPY could be associated with its antioxidant capacity.

In conclusion, in the present study, we show that 40 mg/kg is the therapeutic trypanocidal dose of Nfx in NMRI mice with acute Chagas disease, while the 10 mg/kg dose is subtherapeutic and has little utility in preventing or reversing Chagas cardiomyopathy development in an NMRI murine model. The side effects observed in healthy mice are mild; therefore, in NMRI mice, severe side effects may be related to drug metabolism by persistent inflammatory cells. DPY enhanced the trypanocidal effect of Nfx, transforming subtherapeutic doses to therapeutic levels, and this effect could be related to their trypanocidal capacity and its mechanism of action related to the prevention or reversal of pathophysiological phenomena related to microvascular theory. Because of their anti-inflammatory, immunomodulatory and antioxidant effects, these medications may improve cardiac function.

The results obtained here in NMRI mice should be interpreted with caution when considering clinical implications in humans with Chagas disease. For example, the 10 mg/kg Nfx dose is subtherapeutic in mice, but this is not applicable for other species, including humans, because the same drug dose will produce different blood levels and tissue concentrations in different species because the bioavailability and biodistribution of the drug differ between different species.

Notwithstanding, given the extensive experience that medical doctors have regarding the clinical use of DPY, its wide therapeutic window, safety and mechanism of action make it an interesting drug that should be tested as an adjunct drug in the treatment of Chagas disease in chronic animal models and then its value assessed in moving on to clinical trials.
